# Kinetics and Dynamics of the Stiff and Flexible Tines with the Duckfoot and the Coulter after Impact with Stones Embedded in Compacted Soil. Part II

**DOI:** 10.3390/ma15041351

**Published:** 2022-02-11

**Authors:** Aleksander Lisowski, Adam Świętochowski, Magdalena Dąbrowska, Jacek Klonowski, Tomasz Nowakowski, Jarosław Chlebowski, Samuel Ferré, Martin Roberge

**Affiliations:** 1 Department of Biosystems Engineering, Institute of Mechanical Engineering, Warsaw University of Life Sciences, Nowoursynowska 166, 02-787 Warsaw, Poland; aleksander_lisowski@sggw.edu.pl (A.L.); magdalena_dabrowska@sggw.edu.pl (M.D.); jacek_klonowski@sggw.edu.pl (J.K.); tomasz_nowakowski@sggw.edu.pl (T.N.); jaroslaw_chlebowski@sggw.edu.pl (J.C.); 2 Soil & Crop Modelling Team, CNH Industrial Canada Ltd., 1000 71st Street East, Saskatoon, SK S7P 0A3, Canada; samuel.ferre@cnhind.com (S.F.); martin.roberge@cnhind.com (M.R.); 3 Chemical & Biological Engineering Department, 57 Campus Drive, University of Saskatchewan, Saskatoon, SK S7N 5A9, Canada

**Keywords:** specific acceleration, specific force, force direction, force tilt angle, mechanical engineering

## Abstract

The kinetics and dynamics of the stiff and flexible tines with the duckfoot and the coulter after impact with stones embedded in compacted soil were examined. The beak of the duckfoot was positioned in the axis of the row of stones embedded in the soil at the depth of stones thickness. The coulter covered the stone or impact the edge of the stone halfway along its length. The tools worked at a speed of 0.83–2.22 m·s^−1^ and a working depth of 0.05–0.10 m. The results of specific parameters were compared to the response of the tools to loads in soil without stones. For both soil conditions, the kinetics of the flexible tine was 24 times more reactive, and the dynamic loads were two times lower than for the stiff tine. The responses of both tines were suppressed along with the working depth because of the more favorable place of impact of the duckfoot beak with the stone. Along with the working speed, for a stiff tine, the specific accelerations decreased significantly, by ten times, and the specific forces increased slightly, by 1.6 times. Among the two systems of setting the coulter, the impact of the cutting edge of the coulter with the stone in the middle of its length was more unfavorable than the work of the coulter covering the stone.

## 1. Introduction

When designing cultivation tools, assumptions are made considering their purpose, and in particular, it is necessary to take into account the structure of the soil and the share of stones in the soil [1]. The fastening elements for tools working in soil must be of adequate strength, which is achieved by selecting a material with high mechanical properties or increasing the cross-section of the shank. These improvements result in higher production costs and a greater weight of the machine, respectively. In order to reduce these inconveniences, the shanks fastening the working elements working in stony soil are equipped with appropriate protection against sudden overload. Creating the optimal design of working units requires knowledge about the resistances generated by the soil and other objects in the soil including stones. At the same time, it is necessary to take into account the requirement of effective cutting of weeds and loosening the soil by the working elements. One such solution is the use of flexible tines in cultivators instead of stiff ones, because both horizontal and vertical forces acting on the cultivating tool decrease with the increase of the oscillation frequency [2]. Obtaining the optimum performance of the tool is achieved by loosening a large volume of soil with an acceptable cutting force [3] while maintaining the correct relationship between the draught force and the loosened area [4]. The increase in volume of soil pores in the disturbed area depends on the nature of the generated stresses and the initial condition of the soil [5].

The effects of improving the soil structure as well as loosening and mixing the soil depend on the type of tool and its geometry. These relationships can be achieved by understanding the relationship between tool type and soil loosening results. This problem is increased when the working element impacts an obstacle in the soil. In the case of small root obstacles, the working elements are exposed to only slightly increased resistance. Rotating elements, even if they are passive elements; e.g., discs blades, deal better with root obstacles and plant remains. These advantages are clearly visible during work on heavily weeded or overgrown fields where the top layer of soil is covered with roots of plant debris [6]. The work of loosening tools is complicated by a large number of stones and in the case of forest soils also by stumps, roots of trees, and shrubs [7]. In the comparative studies, it was found that in the frontal interaction with the stump, the reinforced weeding tool with duckfoot had a similar reaction to the cultivator disc; and with the side effect, the power demand was similar to that of standard weeders [8]. Registered damage to cultivation tools working in stony soils or in the conditions of forest clearing requires changes in design, strength, and geometric parameters [8]. In subsequent studies [9], four types of tools were used, i.e., disc plow, cultivator disc, sweep tool, subsoiling tool, and it was found that the greatest reactions were recorded during the frontal defeat of a stump by a disc plow; and in the case of a side impact with a stump, by a subsoiler. To develop practical tools working in soils with hard obstacles, obstacle–tool interactions and the ability to overcome various obstacles must be studied.

Overcoming soil stresses requires inputs from the tool that are characterized by specific resistance (in N·m^−2^). The lower the specific resistance value is, the better the performance of the tool will be [10]. There is no proposal in the literature to evaluate the tool working in stony soil with the reaction of the tool after impacting the stone.

The soil–tool interactions can be represented by the forces generated at the soil–tool interface in a 3D plane (draught, vertical, and lateral forces) and the displacement of soil particles [11]. The available literature lacks information on the tool–stone interaction and it will be necessary to propose a modified parameter that will allow the comparison of specific resistances taking into account the mass of the stone.

The aim of the study was to (1) investigate the responses of the flexible and stiff tines with duckfoots and a disc working in soil with and without stones at different depth *a* and working speed *v*; (2) determination of specific accelerations (*a_xm_*, *a_ym_*, *a_zm_*) and specific forces (*F_m_*, *F_xm_*, *F_ym_*, *F_zm_*) in the 3D plane acting on the tools; (3) determination of the directional coefficient *k_Fxy_* and the modulus of the angle *α_Fxy_* for resultant specific force *F_xym_* in the horizontal plane *xy*; (4) determination of the directional coefficients (*k_Fx_*, *k_Fz_*, *k_Fy_*) and the angle modulus (*α_Fx_*, *α_Fz_*, *α_Fy_*) of the resultant force *F_m_* in the 3D plane, with respect to the resultant on the horizontal *xy*, vertical (full) *xz*, vertical frontal *yz* planes, respectively.

In order to reduce random factors, the tests were carried out in controlled soil bin conditions at various depths and working speeds of the tools [12]. The variable factors were as follow: the type of tool (stiff tine with duckfoot, flexible tine with duckfoot, cultivator disc); soil condition (without and with stones); working depth *a*; working speed *v* of the tool. Experimental data were processed by analysis of variance and correlation. Based on the results of these analyses, the impact of each factor on the criteria parameters was assessed, i.e., acceleration, force, directional coefficients, and tilt angles of these forces. Because of the large number of criteria parameters (15), their selection was carried out using the feature correlation analysis increasing the perception of information processing [13].

The novelty of this article was to investigate for the first time the responses of cultivation tools after impact with stones and to develop specific kinetic and dynamic indicators related to the mass of stone and the mass of soil with an equivalent volume of stone, characterizing the response to impact loads of selected tools working in soil with and without stones.

## 2. Materials and Methods

### 2.1. Fastening Elements and Tools

**Figure 1:**
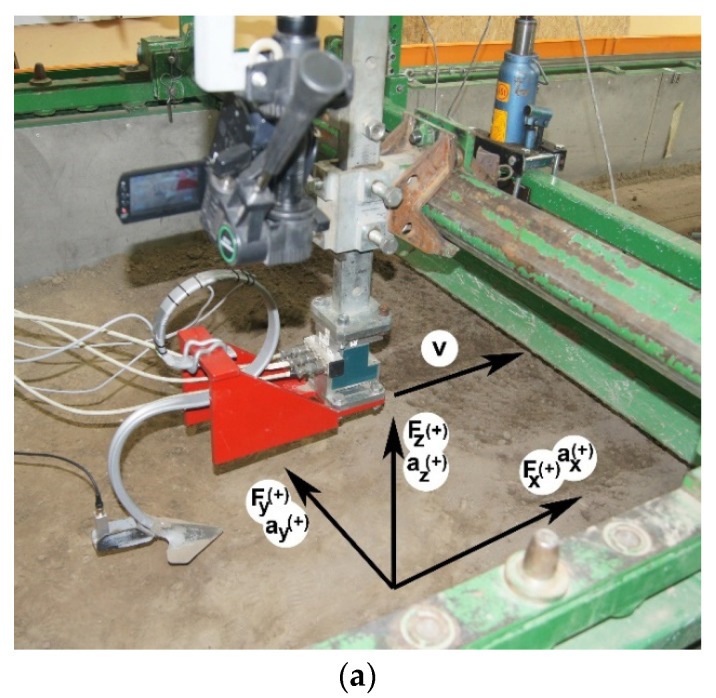

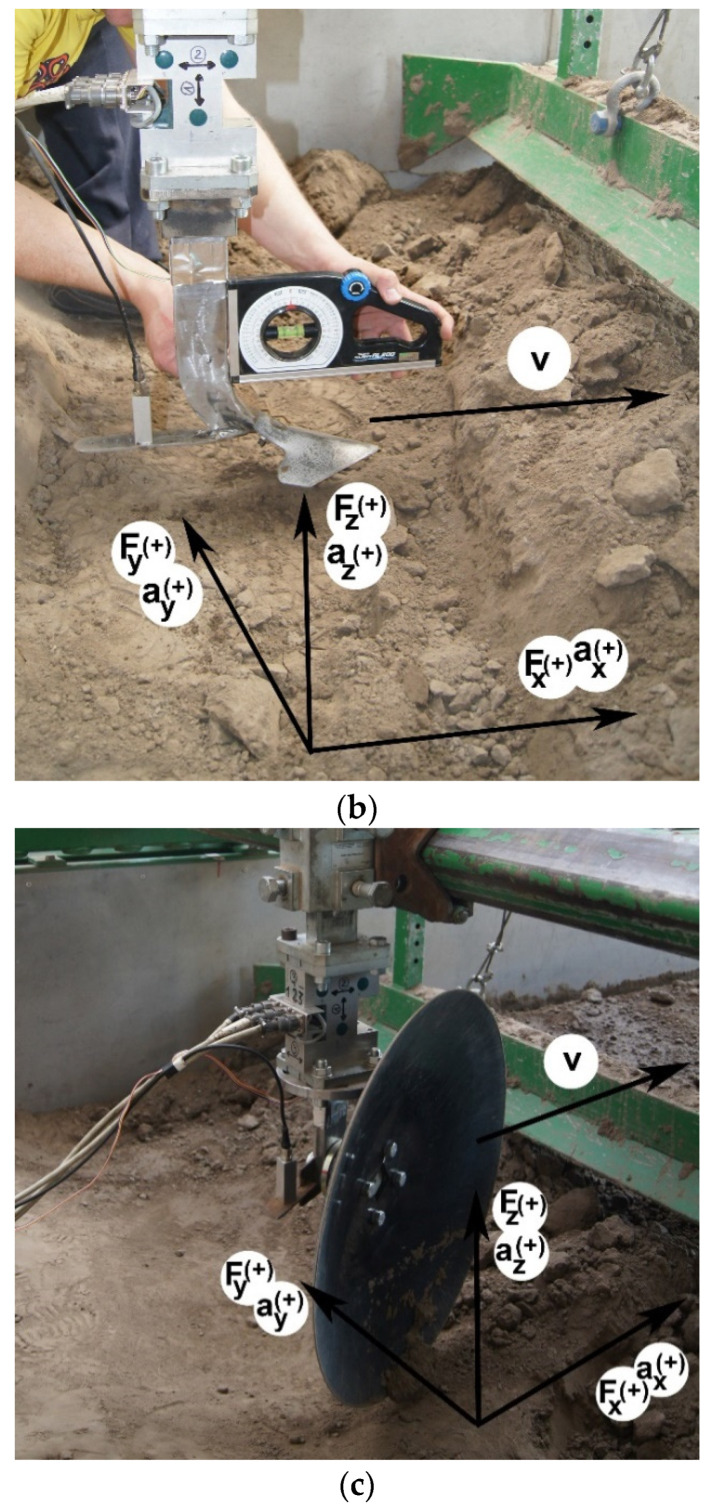
Tested working elements: (**a**) VCO flexible tine with duckfoot, (**b**) stiff tine with duckfoot, (**c**) shaft with coulter set at the 23° rake angle. The figure shows the directions of accelerations and forces acting on the fastening: *a_x_*, *a_y_*, *a_z_*—acceleration: draught, lateral, and vertical, respectively; *F_x_*, *F_y_*, *F_z_*—forces: draught, lateral, and vertical, respectively; (+)—the assumed positive direction of the vector; *v*—working speed.

The VCO flexible and stiff tines with a 135 mm wide duckfoot and a 10 × 60 mm shaft with a disc of 560 mm in diameter were examined (Figure 1). More detailed information can be found in the first part of the article [14].

Forces in tools: draught *F_x_*, lateral *F_y_*, and vertical *F_z_* were measured using a strain gauge three-way force sensor (CS3D, ZEPWN, Marki, Poland). The accelerations of tools in the same directions were measured using a vibration sensor MA 24.01L N:628/10 3 × 160 m·s^−^^2^ (APEK, Warsaw, Poland). The three-way acceleration sensor was mounted vertically to the rear part of the working element in a plane perpendicular to the direction of movement and the ground. The position of the force and vibration sensors was checked with a digital level DNM 60L from Bosch, with accuracy of 0.1°.

The measuring system consisted of a laptop with the test.commander program (Gantner Instruments, Schruns, Austria) for the configuration of the measuring system and data recording. The system was connected to the test controller Q.station101 and measurement modules Q.A107, Q.A108, Q.A116 (Gantner Instruments, Schruns, Austria) for the control, acquisition, and manipulation of measured physical quantities. The sampling frequency of the forces and accelerations was 1000 Hz.

### 2.2. Conditions of Tests

Tests were carried out in the soil bin belonging to the Department of Biosystems Engineering, Institute of Mechanical Engineering, Warsaw University of Life Sciences. The soil bin measuring 10 × 2 × 1 m was filled up to 0.6 m with fine loamy soil (PN-R-04033) with a dry density 1535 ± 11 kg·m^−3^ and moisture content 9.4 ± 0.3% wb. Soil composition was as follow: clay content (<0.002 mm) 2%; silt content (0.002–0.05 mm) 36%; sand content (>0.05 mm) 62%. Mechanical properties of the soil: angle of internal friction 37°; angle of soil-metal friction 24°; cohesion 17 kPa; adhesion 10 kPa. Soil compaction was 421 ± 13 kPa was measured using the electronic penetrometer Penetrologger model 06.15.SA (Eijkelkamp Soil & Water, Giesbeck, Zevenaar, Netherlands) equipped with a cone of apex angle 30° and a base diameter of 20.60 mm.

The soil was loosened to a depth of 0.22 ± 0.02 m with a subsoilers tines and after levelling the surface, it was compacted with a roller weighing 360 kg at single passing in both directions of the soil bin.

**Figure 2:**
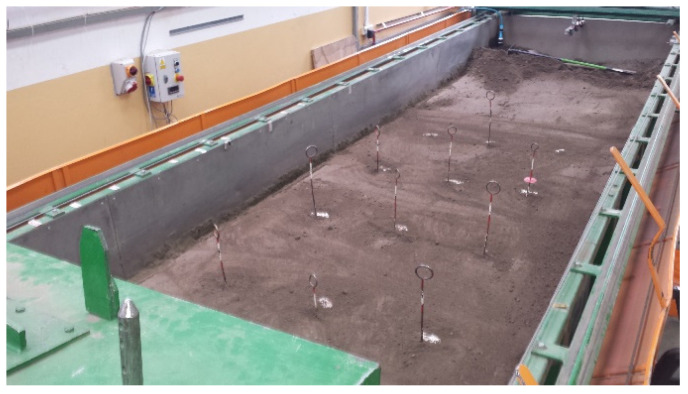
Compacted soil with stones embedded in three rows at a distance of 0.33 m and five columns at a distance of 0.80 m to the trials with coulter, whose edge was set in the axis of the stones row impacting the stones in the middle of their length.

The stones weighing 2.52–4.72 kg, with sphericity of 0.785–0.900 and average density 2831 kg·m^−3^ [14] were embedded in the soil in 3, 4, or 5 rows in distances 0.50, 0.40, and 0.33 m, respectively for flexible tine, stiff tine, and coulter, respectively, to prevent stone and soil interaction between adjacent tool passes. The distance of the stones between the columns in rows was equal and amounted to 0.80 m (Figure 2). The soil with embedded stones was compacted by a roller with weights with a total weight of 520 kg.

The duckfoot attached to the flexible and stiff tine was set along the axis of the stone row. The coulter covered the stones at one mount and at the other its edge was in the axis of the stones row and impact the stone in the middle of its length.

The working depths of the duckfoot with the flexible tine were 0.05 and 0.07 m and with stiff tine 0.07 and 0.10 m. The coulter worked at the depth of 0.07 and 0.10 m. The speeds of tines with duckfoot and the coulter covering the stone were 0.83, 1.67, and 2.22 m·s^−1^ and of coulter in the axis of the stones row 0.83 and 1.67 m·s^−1^.

For comparative purposes, tool tests on soil without stones were also carried out.

### 2.3. Kinetics and Dynamics of Movement of Fastening Elements with Tools

**Figure 3:**
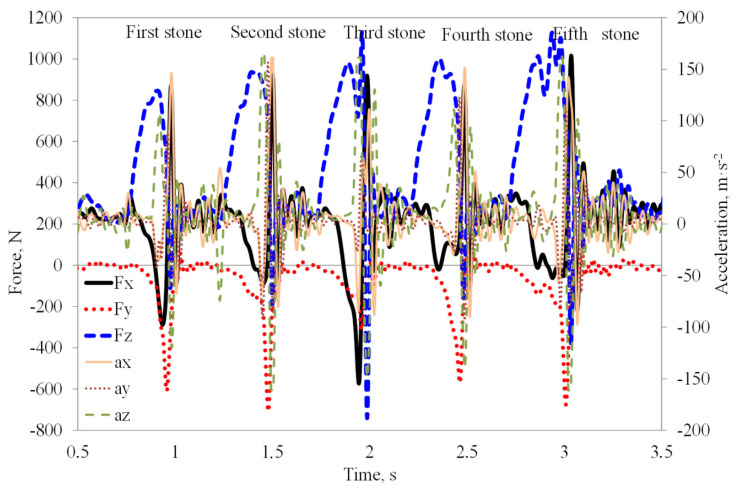
An example of recorded forces: draught *F_x_*, lateral *F_y_*, and vertical *F_z_* and accelerations *a_x_*, *a_y_*, and *a_z_* in three mutually perpendicular directions during work of flexible tine with duckfoot at a depth of 0.07 m and speed 1.67 m·s^−1^ (repeated peaks indicate a kinetic and dynamic responses when the tool impact a stone).

The recorded signals were converted into real values of acceleration and forces of tools using the inverse regression functions obtained from sensor scaling introduced into the analytical software. For the analysis of the parameters characterizing the responses of the tools after a stone impact, 20 measurement points around the extreme value were selected (Figure 3).

As the mass of the stones was different, in order to compare the test results the concept of specific force and specific acceleration related to the mass of the stone was introduced.

In order to compare the specific force and specific acceleration in the impact of the tool with the stone with the response of the tool working in soil without stones, it was assumed that the volume of soil *V_e_* displaced by the tool was equal to the volume of stone V_s_. The mass of soil taken over by this volume was calculated from Equation (1).

(1) $$m_{e}=\frac{m_{s}}{\rho_{s}}\frac{\rho_{e}}{1-MC/100},$$

where: *m_e_*, *m_s_* is a mass of soil and stone, respectively, kg; *ρ_e_*, *ρ_s_* is a density of DM soil and stone, respectively, kg·m^−3^; *MC* is a moisture content of soil relative to the wet substance, %.

**Figure 4:**
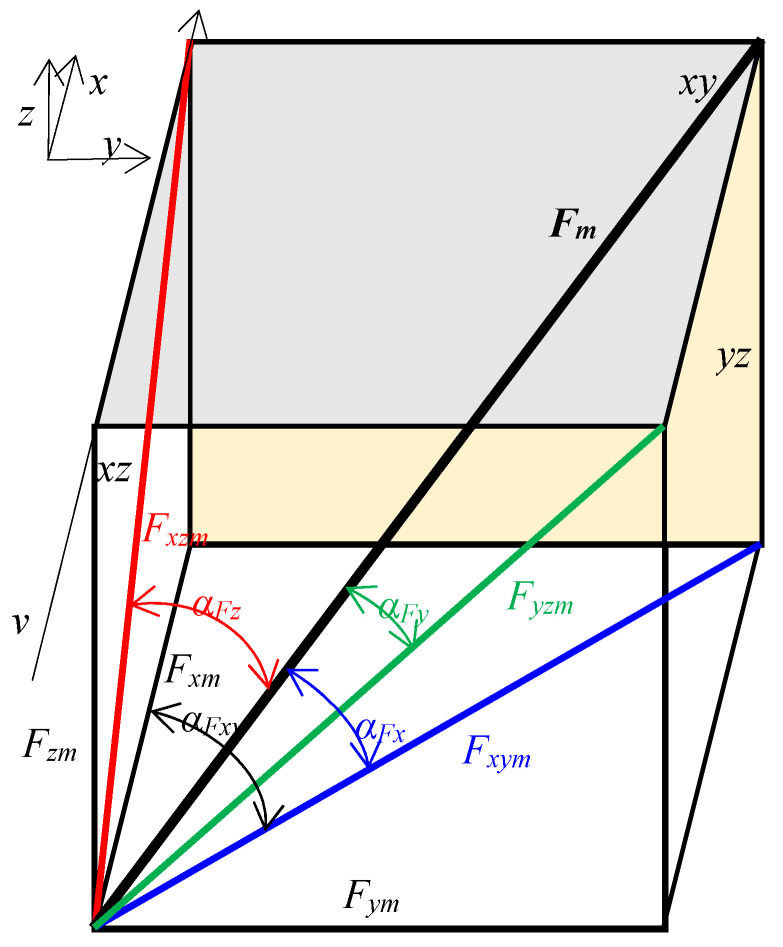
Forces of tools and their tilt angles in the 3D plane. *F_m_*—specific resultant force in the 3D plane; *F_xm_*, *F_ym_*, *F_zm_*—specific forces: draught, lateral, and vertical, respectively; *F_xym_*—resultant specific force in the horizontal plane *xy*; *α_Fx_*, *α_Fz_*, *α_Fy_*—modulus of the tilt angle of resultant force *F_m_* in the 3D plane, relative to the resultant force in the *xy* horizontal plane, *xz* vertical plane (face), and *yz* vertical frontal plane, respectively; *α_Fxy_*—modulus of the tilt angle of force in the *xy* horizontal plane; *x*, *y*, *z*—axis, in the front, lateral, and vertical movement, respectively; *xy*, *xz*, *yz*,—the plane: horizontal (upper), vertical (side), vertical frontal, respectively; *v*—working speed and the direction of tool movement.

Specific forces and specific accelerations were calculated from Equations (2) and (3), respectively.

(2) $$F_{jm}=\frac{\sum_{i=1}^{n}F_{ji}}{nm_{o}},\;\mathrm{where}\;j=x,\;y,\;z;\;n=20;\;o=s,\;e,$$

(3) $$a_{jm}=\frac{\sum_{i=1}^{n}a_{ji}}{nm_{o}},\;\mathrm{where}\;j=x,\;y,\;z;\;n=20;\;o=s,\;e,$$

where: *F_jm_* is a specific force in the direction of considered axis in the 3D plane (Figure 4), N·kg^−1^; *F_ji_* is the momentary force of the tool in impact with a stone in the direction of considered axis in the 3D plane, N; *a_jm_* is a specific acceleration in the direction of considered axis in the 3D plane, m·s^−2^·kg^−1^; *a_ji_* is a momentary acceleration of the tool in impact with a stone in the direction of considered axis in the 3D plane, m·s^−2^; *m_o_* is a mass, kg; *o* means an object; *s* means a stone; *e* means a soil; *j* means an axis *x*, *y*, *z* in the 3D plane; *x*, *y*, *z* are the axes in the front, lateral and vertical movement, respectively; *i* is the next measuring point; *n* is the number of measurement points, assumed *n* = 20.

The direction of the tool forces depended on the place of impact with the stone. The forces acting on the tool were characterized by directional coefficients defining the direction of the force vector, which assumed positive and negative values, and by the tilt angles of these forces, but as modules of their values. The directional coefficients of forces will allow the analysis of the asymmetry of the tool response to stone impact or soil resistance. The modulus of the angle because of the symmetry property of the module will allow to determine the absolute direction of the tool response.

The directional coefficient of the force acting in the *xy* horizontal plane in relation to the direction of the tool movement (Figure 4) was calculated from the Equation (4).

(4) $$k_{Fxy}=\frac{F_{ym}}{F_{xm}}$$

where: *k_Fxy_* is the directional coefficient of the specific force *F_xym_* in the horizontal plane *xy*; *F_xm_*, *F_ym_* are specific forces: draught and lateral, respectively, N·kg^−1^.

The modulus of the tilt angle of force in the *xy* horizontal plane in relation to the tool movement (Figure 4) was calculated from Equation (5).

(5) $$\alpha_{Fxy}=\left|\tan^{-1}\frac{F_{ym}}{F_{xm}}\right|$$

where: *α_Fxy_* is modulus of the tilt angle of force in the *xy* horizontal plane, °; *F_xm_*, *F_ym_* are specific forces: draught and lateral, respectively, N·kg^−1^.

The directional coefficient of the specific resultant force in the 3D plane and modules of tilt angles of this force (Figure 4) were calculated from the Equations (6) and (7), respectively.

(6) $$k_{Fx}=\frac{F_{zm}}{\sqrt{F_{xm}^{2}+F_{ym}^{2}}},\;k_{Fy}=\frac{F_{xm}}{\sqrt{F_{zm}^{2}+F_{ym}^{2}}},\;k_{Fz}=\frac{F_{ym}}{\sqrt{F_{xm}^{2}+F_{zm}^{2}}},$$

(7) $$\alpha_{Fx}=\left|\tan^{-1}\frac{F_{zm}}{\sqrt{F_{xm}^{2}+F_{ym}^{2}}}\right|,\;\alpha_{Fy}=\left|\tan^{-1}\frac{F_{xm}}{\sqrt{F_{zm}^{2}+F_{ym}^{2}}}\right|,\;\alpha_{Fz}=\left|\tan^{-1}\frac{F_{ym}}{\sqrt{F_{xm}^{2}+F_{zm}^{2}}}\right|,$$

where: *k_Fx_*, *k_Fz_*, *k_Fy_* is the directional coefficient of the specific resultant force *F_m_* in the 3D plane, relative to the resultant force on the *xy* horizontal plane, *xz* vertical plane (face), *yz* vertical frontal plane; *F_xm_*, *F_ym_*, *F_zm_* are specific forces: draught, lateral, and vertical, respectively, N·kg^−1^; *α_Fx_*, *α_Fz_*, *α_Fy_* are modules of the tilt angle of resultant force *F_m_* in the 3D plane, relative to the resultant force in the *xy* horizontal plane, *xz* vertical plane (face), and *yz* vertical frontal plane, respectively, °; *x*, *y*, *z* are axes, in the front, lateral, and vertical movement, respectively.

The correctness of calculations of the modules of tilt angles of forces in the 3D plane was checked on the basis of Equation (8).

(8) $$\cos^{2}\alpha_{Fx}+\cos^{2}\alpha_{Fy}+\cos^{2}\alpha_{Fz}=2$$

where *α_Fx_*, *α_Fy_*, *α_Fz_* are the modules of the tilt angle of resultant force in the 3D plane in the horizontal, lateral, and vertical directions, °.

### 2.4. Selection of Kinetic and Dynamic Parameters

The selection of kinetic and dynamic parameters was carried out using the feature correlation method; therefore, eliminating parameters whose numerical values are strongly correlated with each other. The purpose of this selection is to limit the criteria parameters towards the suggested number 7 ± 2 [13] increasing human perception of information processing.

The input to the analysis was the matrix of correlation coefficients **R** determined for the analyzed features for which the inverse matrix **R**^−1^, **R**^−1^ = **R**^T^/det(**R**) was created, where **R**^T^ is a transposed matrix and det (**R**) is a determinant of the **R** matrix (det(**R**) ≠ 0). For the parameter strongly correlated with the others, the elements of **R**^−1^ exceeded the value of 10. It meant bad numerical condition of the matrix **R**. The parameter was removed from the original set when the diagonal value was ≥10. Parameters were eliminated cyclically. After discarding one parameter strongly correlated with others, the procedure was repeated. The correctness of the obtained results was checked by determining the unit matrix **I** = **R**·**R**^−1^.

The feature correlation coefficient matrices were determined using the Statistica v.13.3 software. The calculations of the determinant of the matrix **R** (det(**R**)), the inverse matrix **R**^−1^, and the unit matrix **I** were performed using an Excel spreadsheet.

### 2.5. Statistical Analysis

The input matrix of the correlation coefficients was determined on the basis of four factors and 15 kinetic and dynamic parameters of the movement of the fastening elements with the cultivation tools.

On the basis of statistical analysis; the influence of the tool type, soil condition, depth *a*, and working speed *v* on acceleration and specific forces, as well as coefficients and tilt angles of forces acting on tools working in soil with and without stones were examined.

The statistical analysis of the results was performed using the Statistica v.13.3 software (StatSoft Poland Ltd., Cracow, Poland). Detailed analysis for mean values was performed with the Tukey test. The significance of differences between mean values was checked at the significance level *p* = 0.05.

## 3. Results and Discussion

### 3.1. Feature Correlation Matrix

Using the feature correlation method, six parameters were removed from the initially proposed parameters, which were characterized by very high values of the r Pearson correlation coefficients, including the tilt angle *α_Fz_* of resultant force *F_m_* in the 3D plane (correlation with *k_Fz_*, r = 0.992); specific resultant force *F_m_* in the 3D plane (correlation with *F_xm_*, r = 0.831); directional coefficient *k_Fz_* of resultant force *F_m_* in the 3D plane relative to the resultant force on the *xz* plane (correlation with *α_Fxy_*, r = 0.899); tilt angle of force *α_Fxy_* in the horizontal plane *xy* (correlation with *k_Fxy_*, r = 0.979); tilt angle *α_Fy_* of resultant force *F_m_* in the 3D plane (correlation with *k_Fxy_*, r = −0.762); specific acceleration *a_xm_* in the direction of tools movement (correlation with *a_zm_*, r = 0.911). After the features were selected, there were 9 parameters that characterize the kinetics and dynamics of tool movement in soil with and without stones (Table 1). Table 1 also includes factors as decision variables that could correlate with other features. The results of the correlation coefficient matrix will be successively described when analyzing individual criteria parameters.

### 3.2. Specific Accelerations of Tools in the Vertical a_zm_ and Lateral a_ym_ Directions

**Figure 5:**
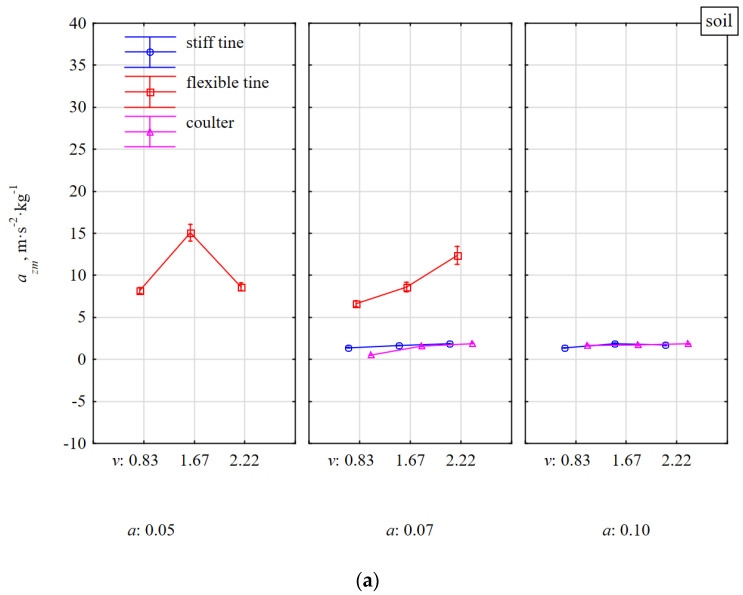

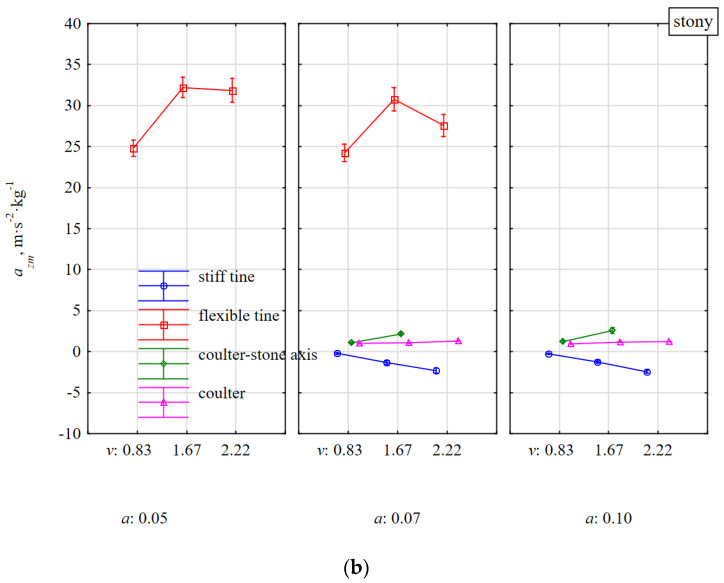
Specific vertical acceleration *a_zm_* of tools under the influence of reaction to soil resistance without stones (**a**) and with stones (**b**). Tools working at different depths *a* and at different working speeds *v*.

Because of the aforementioned selection of specific acceleration in the direction of tool movement *a_xm_*, a more detailed analysis took into account specific accelerations in the vertical *a_zm_* and lateral *a_ym_* directions. Specific accelerations of tools *a_zm_* and *a_ym_* were statistically significantly different in relation to all examined factors; tool type, soil condition, working depth, and speed, as the *p*-value was in the range 0.0001–0.0332 (Table 2). The reference to the kinetic response of the tools on impact with stones was the behavior of tools working in soil without stones (Figure 5a).

For soil without stones, specific accelerations for all tools in the vertical direction were directed upwards as the values were positive (Table 2). Accelerations *a_zm_* of stiff tine and coulter working in the soil without stones did not depend on the working speed and depth and were within a narrow range of 0.55–1.88 m·s^−^^2^·kg^−1^ (Figure 5a). The flexible tine response was much more dynamic (*a_zm_* = 6.61–15.08 m·s^−^^2^·kg^−1^) and was increased with the working speed and was lower at a greater depth (0.07 m) than at a lower depth (0.05 m). The oscillating behavior of the flexible tine was more ambiguous at a lower than a greater working depth. These oscillations could be the reason for more frequent coming out of flexible tine from the soil and the flexible tine to deflect less back and up. At a greater working depth, the flexible tine in the return movement was suppressed by the larger volume of loose soil, which was compressed by the duckfoot before the tool came into contact with firm soil. Compression of loose soil, prior to cutting hard soil, by a tool that moves forward after it is tilted backward was noticed by Yow and Smith [15].

After impacting a stone, the flexible tine was subjected to very high accelerations *a_zm_*, which were 2.2–3.7 times greater than during work in soil without stones. Taking into account the variability of vibrations and deviations of the flexible tine, the duckfoot working at a depth of 0.05 m impact the stone above the middle of the stone thickness (0.056 m). Under these conditions, the flexible tine after impacting the stone was rapidly deflected back and up and the accumulated energy in the spring was transferred to the tine’s return movement generating greater acceleration *a_zm_*. At a working depth of 0.07 m, the flexible tine impacted the stone just below the center of the stone’s thickness. At the moment of contact of the tool with the stone, the soil in front of the stone was compressed, which contributed to a slight damping of impact and a reduction in the acceleration of the tool.

With an increase in the working speed from 0.83 to 1.67 m·s^−^^1^, accelerations *a_zm_* of the flexible tine after impacting a stone increased by 27–30% and were higher by 8% for greater than shallower working depth (Figure 5b). At higher working speeds, the kinetics of the flexible tine in contact with the stones was more reactive and during work at a speed of 1.67 m·s^−^^1^ at depths of 0.05 and 0.07 m, the acceleration increased to 32.21 and 30.78 m·s^−^^2^·kg^−1^, respectively, then at the speed of 2.22 m·s^−^^1^ they decreased to 31.87 and 27.59 m·s^−^^2^·kg^−1^, respectively. This reduction in acceleration could have resulted from a lower velocity ratio, which is the relation between the amplitude velocity and the speed of the tool movement, whose impact was analyzed with the load of sinusoidal vibrating tools [15].

During the impact of the stiff tine with stones embedded in the compacted soil, the accelerations *a_zm_* were directed downwards and increased significantly with the working speed and only to a small extent depended on the working depth.

During the impact of flexible tines with stones, the specific accelerations in the direction of movement and vertical were 34.9 and 28.6 m·s^−^^2^·kg^−1^, respectively, and for stiff tine they were −12.2 and −1.3 m·s^−^^2^·kg^−^^1^, respectively (directed downwards).

The reaction of coulter covering the stone was more damped than when the coulter was working on soil without stones and was independent of the speed and working depth. At the working speed of 1.67 m·s^−^^1^ when the coulter edge impacted the stone, the acceleration *a_zm_* was twice as high as for the coulter covering the stone and at a lower working speed of 0.83 m·s^−^^1^, the acceleration difference was only 8%.

**Figure 6:**
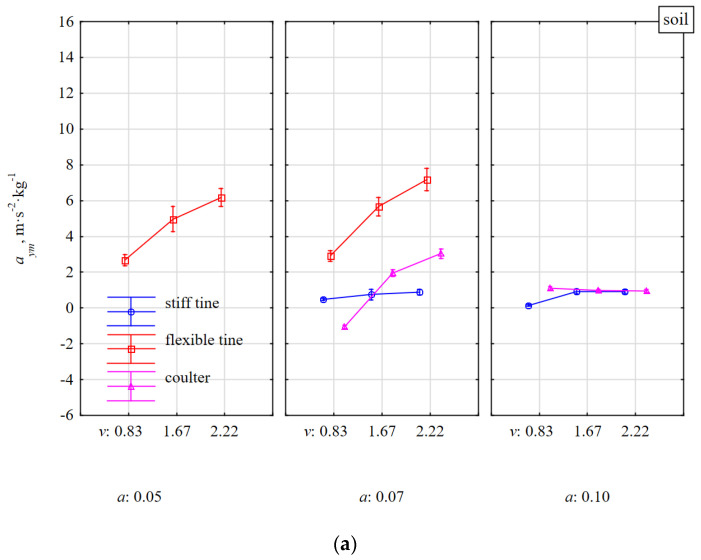

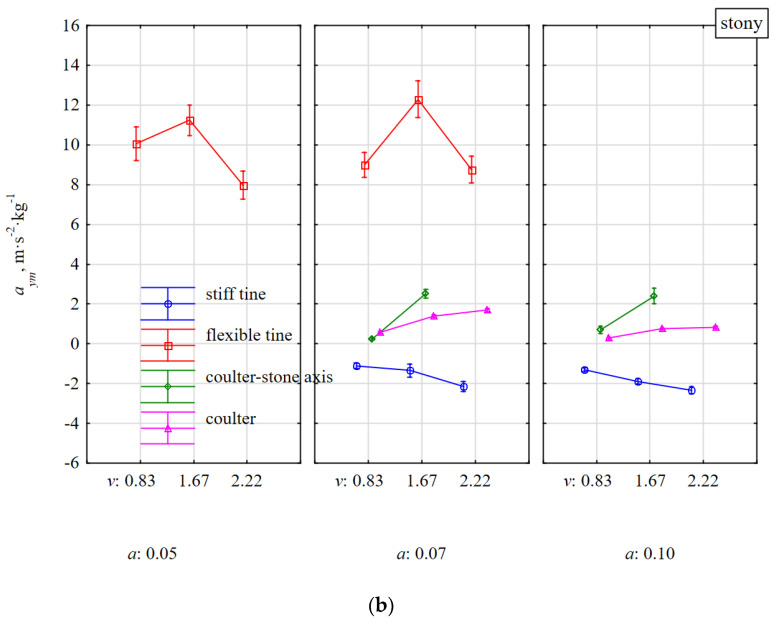
Specific lateral acceleration *a_ym_* of tools under the influence of reaction to soil resistance without stones (**a**) and with stones (**b**). Tools working at different depths *a* and at different working speeds *v*.

The specific accelerations of the tools in the vertical direction *a_zm_* were very highly correlated with the accelerations in the lateral direction *a_ym_*, r = 0.759 (Table 1), but the *a_ym_* were more varied between the tools (Figure 6). The *a_ym_* acceleration values were significantly lower, especially for the flexible tine. Positive values of *a_ym_* indicate a direction of the acceleration to the right and negative values—to the left of the direction of travel (as marked in Figure 4).

The *a_ym_* accelerations of the flexible tine working in soil without stones clearly increased with the working speed and slightly increased with the working depth. The greater the speed and working depth, the greater was the asymmetry of the duckfoot loads. These asymmetrical loads can result from asymmetry of the impacts or geometry of the duckfoot or the attachment of the flexible tine despite careful installation and accurate measurement of the settings. A similar situation occurred for a stiff tine, but with a lower dynamics of changes in relation to the working parameters and less asymmetry (Figure 6).

For the coulter working in the soil without stones at a depth of 0.07 m and at the lowest working speed of 0.83 m·s^−^^1^, the *a_ym_* acceleration was −1.05 m·s^−2^·kg^−^^1^ and was directed to the left and at higher speeds (1.67 and 2.22 m·s^−^^1^) the accelerations increased and were directed to the right in relation to the movement direction and amounted to 1.95 and 3.03 m·s^−2^·kg^−^^1^, respectively. During the coulter operation at a greater working depth (0.10 m), the motion kinetics was stable and did not depend on the working speed, and the average coulter acceleration was 1.01 m·s^−2^·kg^−^^1^.

The reactions of the flexible tine after an impact with a stone in the lateral direction were characterized by the highest *a_ym_* acceleration directed to the right of the movement direction, which was about two times greater than when the flexible tine was working in soil without stones and ranged from 8 to 12 m·s^−2^·kg^−^^1^ (Figure 6b). The stiff tine achieved *a_ym_* acceleration to the left, which increased almost twice in the range of the tested working speed and by 21% when changing the depth from 0.07 to 0.10 m, but the acceleration values were small and ranged from −1.10 to −2.34 m·s^−2^·kg^−^^1^. According to the setting of the coulter at a rake angle of 23° and the movement of the coulter to the left because of the resistance of the deposited soil with the stone to the right of movement direction, the coulter acceleration *a_ym_* was directed to the right, opposite to the movement of the tool. The *a_ym_* acceleration of the coulter covering the stone increased threefold in the range of the movement speed 0.83–2.22 m·s^−1^, but the *a_ym_* values were small and ranged from 0.29 to 1.70 m·s^−2^·kg^−^^1^. When the coulter edge impacted the stone in the middle of its length, a greater kinetic response was showed and at the working speed of 1.67 m·s^−1^, the *a_ym_* acceleration was 2.50 m·s^−2^·kg^−^^1^.

### 3.3. Specific Draught F_xm_, Vertical F_zm_, and Lateral F_ym_ Forces of Tools Working in the Soil with and without Stones

In most cases, specific forces were statistically significantly differentiated with respect to the tested factors, *p* < 0.0001 (Table 2). Only the lateral specific force had statistically insignificant differentiated values for tools working in soil with and without stones (*p* = 0.9636).

**Figure 7:**
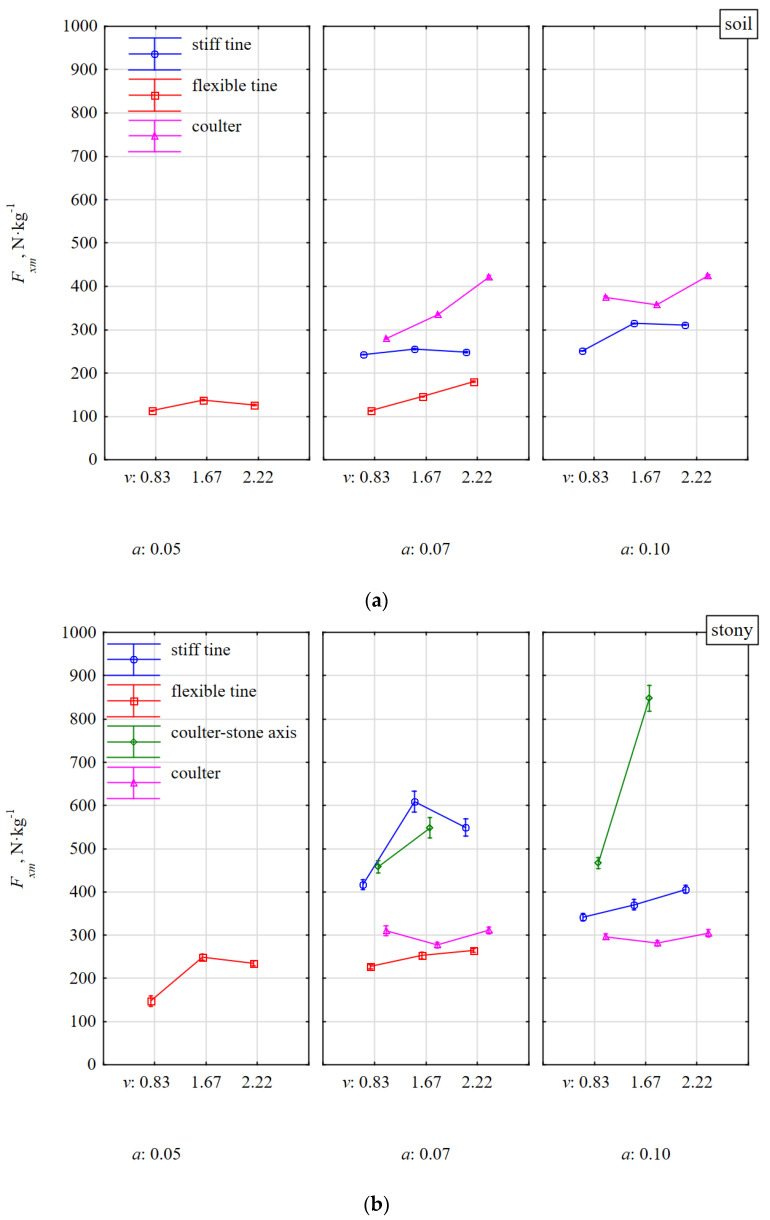
Specific draught force *F_xm_* of tools in response to resistance of soil without stones (**a**) and with stones (**b**). Tools working at different depths *a* and at different working speeds *v*.

The specific draught force *F_xm_* was 70% higher during work of the flexible tine in the soil with stones than without stones (Figure 7) and was the smallest among all the tools. The ranges of mean values for this tine were 113–180 N·kg^−^^1^ and 148–264 N·kg^−^^1^, respectively. The characteristics of changes in this draught force were similar both in terms of speed and working depth.

For the working depth of 0.07 m, the force of the stiff tine after impacting a stone was definitely more dynamic and *F_xm_* increased on average from 248 to 525 N·kg^−^^1^ (by 112%) both in relation to the flexible tine and the condition of the soil; without stones and with stones. These changes in force values are pieces of evidence confirming the current knowledge that a flexible tine has less resistance than a stiff tine (by 60%). In addition, the flexible tine requires more than two times less draught force during an impact with a stone than a stiff tine [16]. Such a large accumulation of an impact for flexible tine with a stone is very important because of the durability and safety of the entire structural system of the machine and the tractor unit.

The rapidity of the force of the stiff tine was a result of the fact that the beak of the duckfoot impacted the stone before the soil wedge made it move. The soil in front of the stone was not sheared, but pressed for a moment as if by a very dull edge of the blade [17] giving additional resistance. While the beak of duckfoot of stiff tine impacted the stone much below the center of the stone’s thickness (0.056 m) at a working depth of 0.10 m, the growth of dynamics of *F_xm_* force was lower and amounted to 27%. Under these conditions, the movement of the stone started with the soil wedge even before the impact of the duckfoot with the stone; therefore, the response of the tool to the impact was less dynamic.

During the impact of flexible tine with stones, the specific forces in the direction of movement and vertical were 229 and −124 N·kg^−^^1^, respectively (directed downwards), and for a stiff tine they were 449 N·kg^−^^1^ and 114 N·kg^−^^1^, respectively.

**Figure 8:**
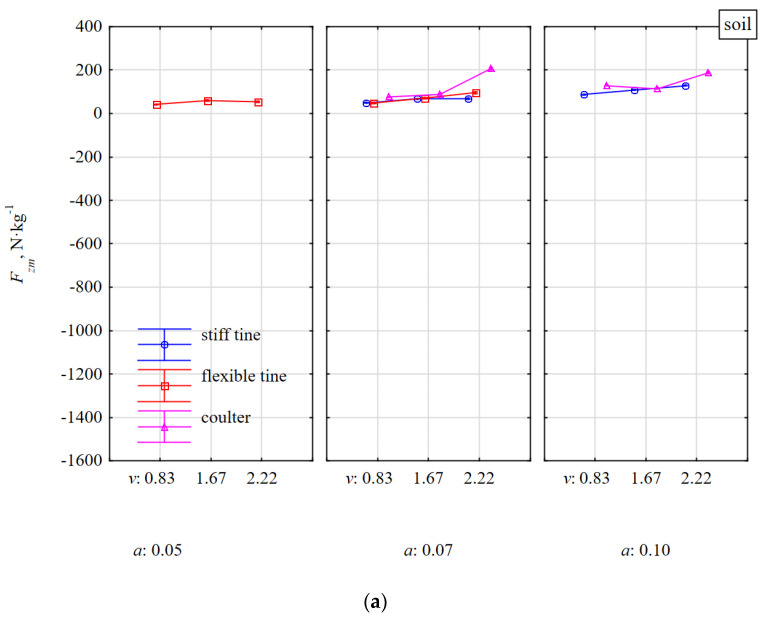

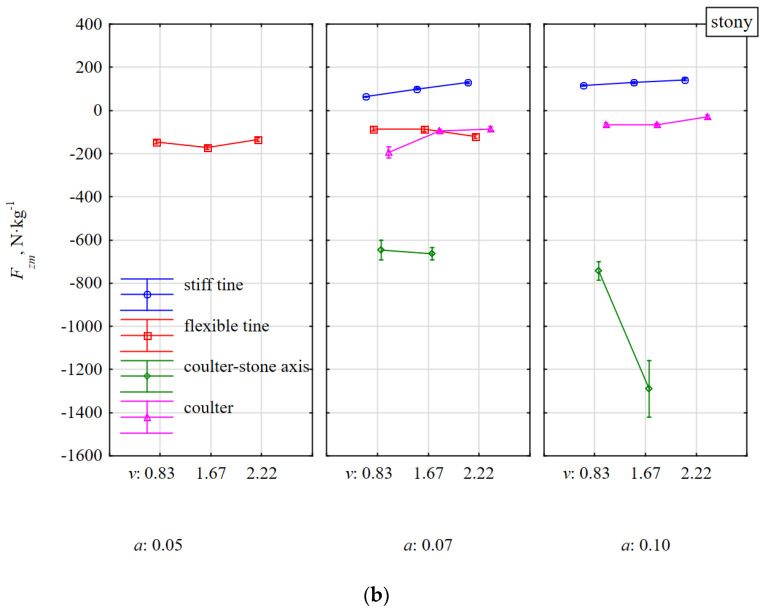
Specific vertical force *F_zm_* of tools in response to resistance of soil without stones (**a**) and with stones (**b**). Tools working at different depths *a* and at different working speeds *v*.

During the work of the coulter covering the stone, the response of the tool was quite stable on both soil with and without stones. The stones were moved with the soil and the specific draught force in these conditions was even lower than for soil without stones. These reductions resulted from the greater density of the stone than the soil and assuming the same volume, it turned into a greater mass of matter that was moved. The work of the coulter impacting the stone in the middle of its length was much more dynamic as the force *F_xm_* increased more than two times in relation to the reaction of the coulter covering the stone. Under these conditions, the coulter rolled over the outer surface of the stone, forcing it into the soil proportionally to the coulter’s working depth. These dynamics of the coulter impacting the stone with respect to the working speed was more noticeable for a greater depth of 0.10 m than for a depth less than 0.07 m and amounted to 82 and 20%, respectively. The vertical specific force *F_zm_* of tools working in soil without stones (Figure 8a) had similar characteristics as the draught force *F_xm_*, but the *F_zm_* force values were almost twice lower than *F_xm_*.

During the work of the flexible tine in the soil with stones, the force *F_zm_* was directed downwards and was independent of the working speed and was greater for a smaller working depth of 0.05 m than for a greater working depth of 0.07 m and was on average −150 and −97 N·kg^−^^1^, respectively (Figure 8b). Because of the elasticity of the tine, the tool tilted, reducing the working depth, which was noted also in previous studies [18]. Therefore, for a shallower depth of 0.05 m, the impact point of the duckfoot beak was above the center of the stone thickness (0.056 m) and the vertical component of the resistance was directed upwards and the tool’s reaction downwards. At a working depth of 0.05 m, the duckfoot attached to the flexible tine more often moved along the outer surface of the stone from above causing its displacement, rotation, or just movement. At a greater working depth of 0.07 m, the stones were moved, but the oscillations and greater resistances related to the depth also made the working depth of the duckfoot shallower, the beak of which impacted the stone near or above the center of the stone’s thickness. It follows that the impact of the tool on a stone embedded in the soil above its center of gravity is the most disadvantageous because of the stone–tool relationship.

The work of the duckfoot attached to the stiff tine was more stable and at the working depths of 0.07 and 0.10 m, the beak of the duckfoot impacted the stone below its thickness center, throwing the stone together with the soil. The force *F_zm_* was directed upwards and depended on the speed and working depth (Figure 8b). Compared to working in soil without stones, the force of the stiff tine *F_zm_* after impacting a stone increased on average by 38%.

During the work of the coulter covering the stone, the force *F_zm_* was directed downwards and was greater for a shallower depth than for a greater working depth and amounted to −124 and −53 N·kg^−1^, respectively. In the range of the working speed of 0.83–2.22 m·s^−1^, the force *F_zm_* decreased more than twice.

The change in the direction of the *F_zm_* force on the coulter covering the stone while working in the soil without stones (upwards) and with stones (downwards) resulted from the impact of the coulter with the stone. At a working depth of 0.07–0.10 m and a significantly larger equivalent diameter of stones equal to 0.123–0.154 m, the concave surface and the cutting edge of the coulter impact the stone. As a result of this impact, the coulter force was directed downwards. As the rake angle of the coulter in relation to the direction of movement was 23° and was close to the optimal value [19], the change of the coulter’s working conditions generated a quick reaction towards the vertical force.

The scrubbing reaction of the convex part on the back of the coulter always creates an upward vertical force while the direction of the vertical force resulting from the passive cutting reaction depends on the coulter rake angle (*α*). At rake angles greater than 90–*δ* (where *δ* is the friction angle between the soil and the coulter surface), the passive reaction force is directed upwards while coulters with smaller rake angles generate a downward force [20]. For the driven coulter, the reaction was directed downward, while for the free-rolling coulter, an upward reaction was also observed [21]. It was explained that this was because of the accumulated volume of soil in front of the coulter as the passive coulter was unable to sufficiently penetrate the soil and shift the soil volume.

Even greater downward force *F_zm_* occurred during the impact of the cutting edge of the coulter in the middle of the stone’s length and for the working depths of 0.07 and 0.10 m, *F_zm_* were on average −655 and −1017 N·kg^−^^1^, respectively. At a greater working depth (0.10 m) when the stone was pressed into the soil to a greater depth, the *F_zm_* force increased significantly at the working speed from −744 to −1290 N·kg^−^^1^. Because of the safety of work, such a high force *F_zm_* at a speed of 1.67 m·s^−^^1^ decided to resign from the tests at an even higher working speed of 2.22 m·s^−^^1^.

**Figure 9:**
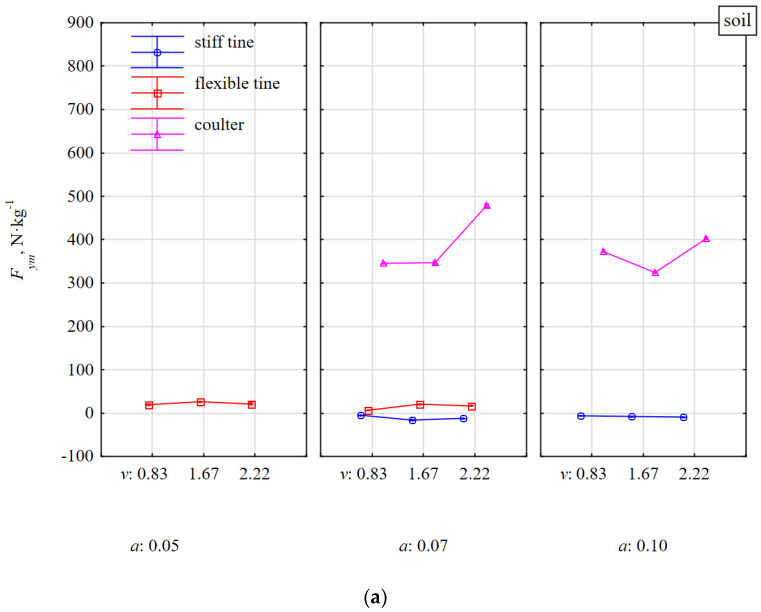

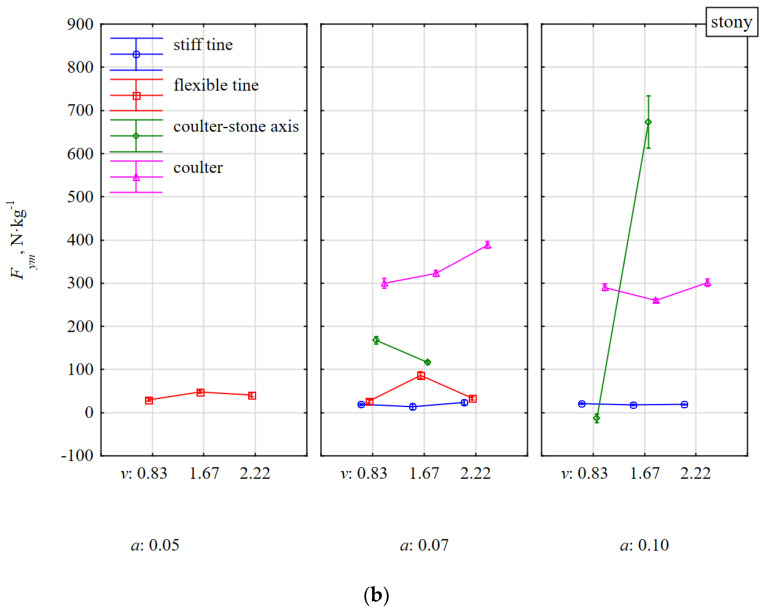
Specific lateral force *F_ym_* of tools as a reaction to the resistance of the soil without stones (**a**) and with stones (**b**). Tools working at different depths *a* and at different working speeds *v*.

Small values of the lateral force *F_ym_* and in different directions for the flexible (6–21 N·kg^−^^1^) and stiff (−5–16 N·kg^−^^1^) tines working in the soil without stones indicate slight asymmetries in the impact and the attachment of duckfoot to the tines (Figure 9a).

During work of these tines in the soil with stones vs. without stones, the *F_ym_* force was only slightly greater (13–87 N·kg^−^^1^) and the direction of the tool forces was to the right (Figure 9b).

Much higher values of the lateral force *F_ym_* were created during the coulter operation both on soil with and without stones. During the work of the coulter covering with stones, the lateral force *F_ym_* was 260–388 N·kg^−^^1^ and was lower than for the coulter working in soil without stones (324–478 N·kg^−^^1^). It was probably related to the impact of the stone with the concave surface of the coulter in its front part and causing a response in the opposite direction to the reaction related to the pressure of the thrown soil with the stone on the coulter.

During work in soil with stones when the cutting edge of the coulter was impacting halfway along the length of the stone, random phenomena occurred that influenced the characteristics of the *F_ym_* lateral force. Most often the coulter pressed the stone into the compacted soil and pushed the stone together with the soil to the right. However, there were also cases that the coulter pressed the stone and pushed it towards the face (to the left). At the coulter working depth of 0.07 m, the *F_ym_* force decreased with the working speed and ranged from 168 to 116 N·kg^−^^1^, for 0.83 and 1.67 m·s^−1^, respectively. Conversely, for the working depth of 0.10 m, *F_ym_* increased with the working speed and were from −13 to 673 N·kg^−^^1^, respectively.

These rapid specific forces recorded in the fastening shanks after the impact of the coulters with the stone occurred in the entire 3D plane (Figure 7b, Figure 8b and Figure 9b). Among the three types of tested tools, disc harrows or disc plows are most vulnerable to damage during an unfavorable axial impact of the coulter edge with stone. Therefore, the coulter mounts should be equipped with safeguards to protect the systems against damage.

### 3.4. The Directional Coefficients k_Fxy_ of Resultant Force F_xy_ in the xy Horizontal Plane, k_Fx_ Total Resultant Force F_m_, Tilted in Relation to the Horizontal Plane xy, k_Fy_ Total Resultant Force F_m_, Tilted in Relations to the Vertical Frontal Plane yz, and the Tilt Angle α_Fx_ of Resultant force F_m_ in Relation to the Horizontal Plane xy

All kinetic parameters were statistically significantly different from the tested factors, *p* = 0.0001–0.0461 (Table 2).

**Figure 10:**
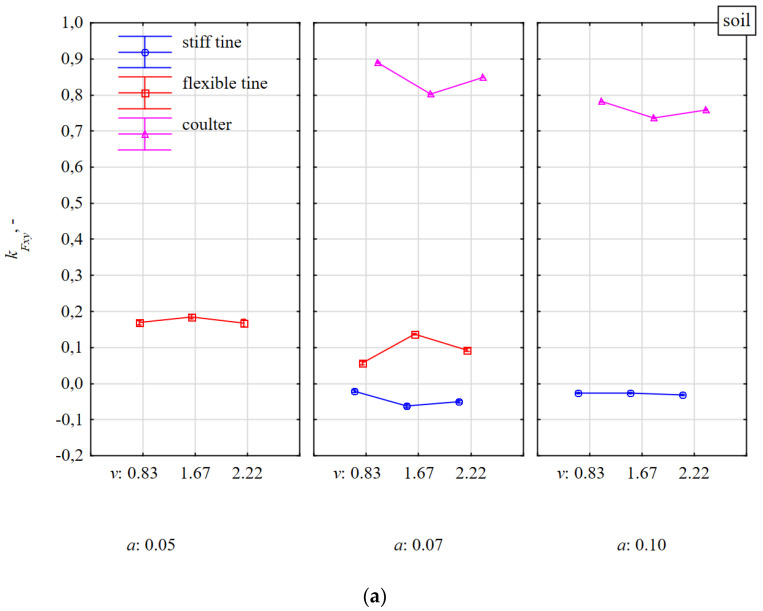

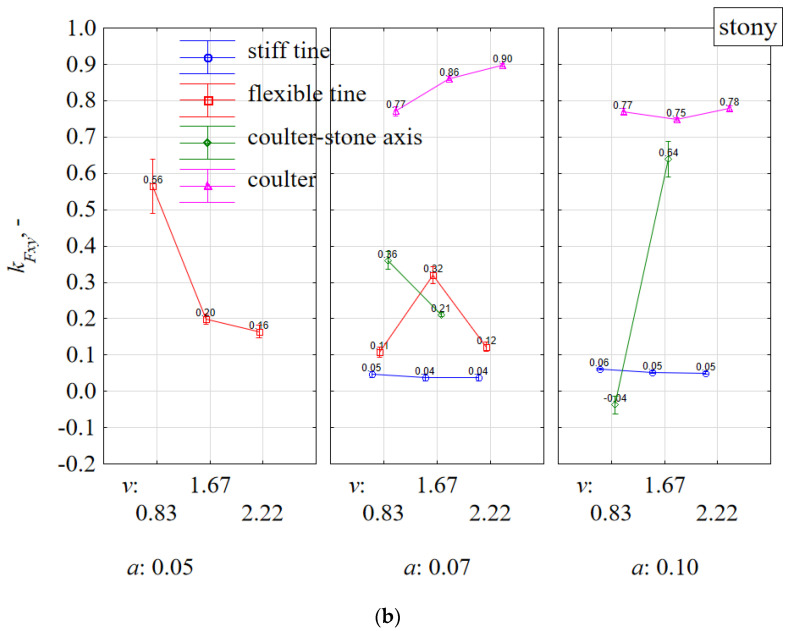
The directional coefficient *k_Fxy_* of resultant force *F_xym_* in the *xy* horizontal plane affecting tools working at different depths *a* and working speeds *v* in a soil without stones (**a**) and with stones (**b**).

The directional coefficient *k_Fxy_* of the resultant force *F_xy_* in the horizontal plane correlated very highly (r = 0.775) with the specific lateral force *F_ym_* (Table 1). Characteristics of the lateral force *F_ym_* decided about the direction of the dynamic response of the tool after the impact with a stone (Figure 9 and Figure 10). Compared to work of tools in soil without stones vs. with stones, the smallest changes occurred for the stiff tine, because *k_Fxy_* = ±0.06; about ± 3.4° (Figure 10). The direction of the resultant force *F_xy_* in the horizontal plane was stable in relation to the working depth and speed.

The direction of the resultant force *F_xym_* for a flexible tine working in soil with stones vs. without stones was more varied, but at the same time ambiguous, which is confirmed by the aforementioned random nature of impacts between a flexible tine and stones.

At the working depth of 0.07 m, the directional coefficient *k_Fxy_* for the coulter covering the stones increased with the working speed, and at the depth of 0.10 m it was stable (Figure 10). During the shallower working depth (0.07 m), the coulter reaction was high as the coulter covered the stone in only 45–57% and the coulter edge impacted the surface of the stone close to its equivalent diameter (0.123–0.154 m). The stones were inside loose, discarded soil. At a working depth of 0.10 m, the concave surface of the coulter covered the stone in 65–81% and the impact of the coulter edge in the stone was insignificant. At this greater working depth, the stone was moved with the soil and was on the outer surface of the loose, discarded soil; therefore, the directional coefficients of the resultant force *F_xym_* for the coulter working in the soil with and without stones were almost identical (0.76 ± 0.02).

The characteristic of the directional coefficient *k_Fxy_* of the resultant force *F_xym_* (Figure 10) as the coulter reaction after the impact with a stone in the middle of its length fully reflects the changes in the specific lateral force *F_ym_* (Figure 9) and previously described interpretation.

**Figure 11:**
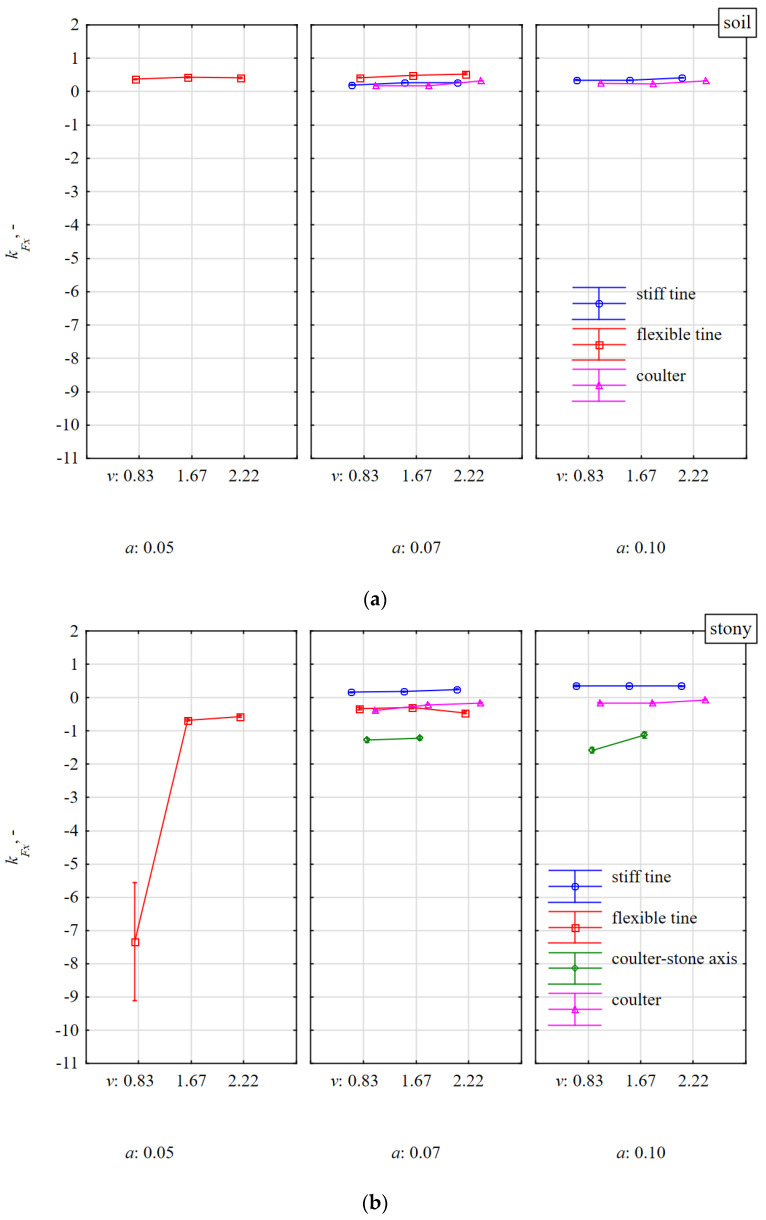
The directional coefficient *k_Fx_* of total resultant force *F_m_*, tilted in relation to the *xy* horizontal plane, affecting tools working at different depths *a* and working speeds *v* in the soil without stones bez (**a**) and with stones (**b**).

The directional coefficient *k_Fx_* of the total resultant force *F_m_* deflected in relation to the *xy* horizontal plane was very stable both during work in soil with and without stones (Figure 11). The only significant deviation occurred for the flexible tine working in stony soil at the working speed of 0.83 m·s^−^^1^ and the working depth of 0.05 m (Figure 11a). Although the tests were performed on 15 stones, the tests were characterized by a significant spread and there were random cases resulting from the shallow duckfoot work and the beak impacting the upper half of the stone.

The largest deviations of the resultant force *F_m_* in relation to the *xy* horizontal plane occurred for the coulter impacting the stone in the middle of its length and ranged from −1.12 to −1.58 (Figure 11b). These deviations of forces are further evidence of significant differences in the values of forces in the 3D plane for the coulter impact with the stone with its simultaneous pressing into the soil.

**Figure 12:**
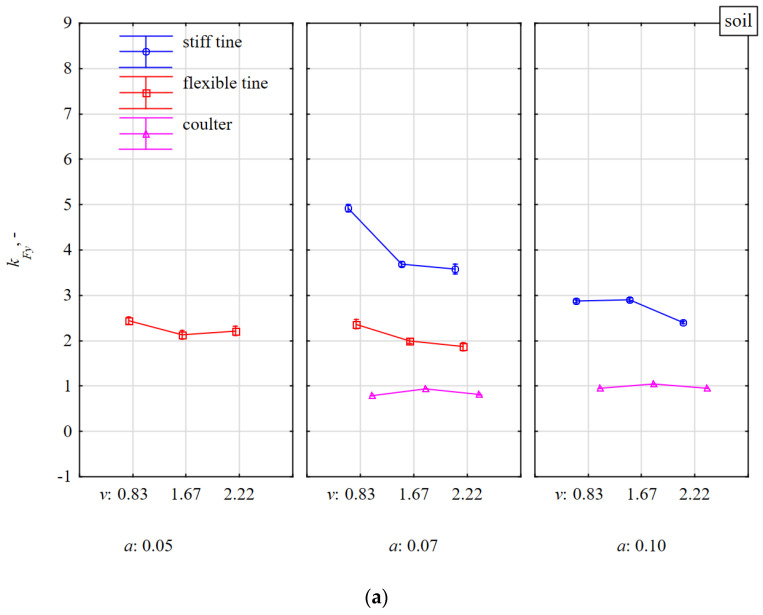

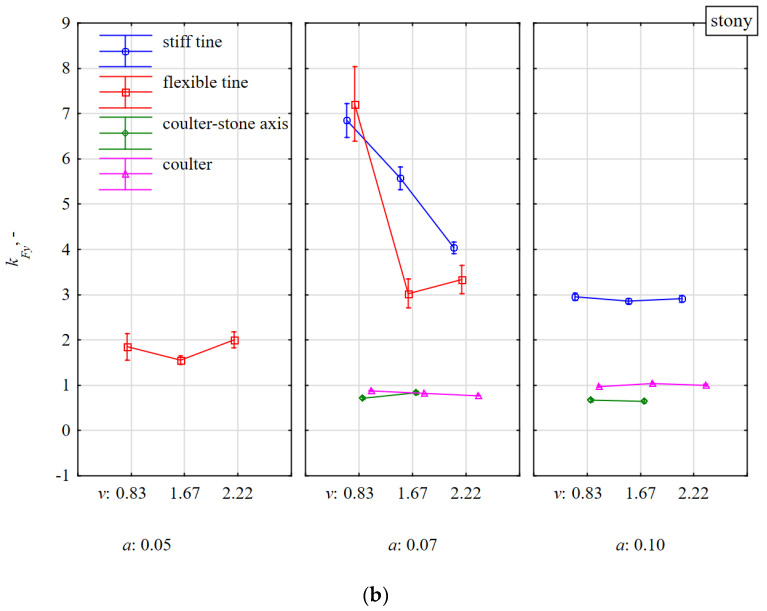
The directional coefficient *k_Fy_* of total resultant force *F_m_*, tilted in relations to the vertical frontal plane *yz*, affecting tools working at different depths *a* and working speeds *v* in the soil without stones bez (**a**) and with stones (**b**).

The directional coefficient *k_Fy_* of the total resultant force *F_m_* deflected in relation to the *yz* lateral plane was more consistent with the force components in the 3D plane than the directional coefficient *k_Fx_* of this *F_m_* force in relation to the horizontal plane. Evidence of this greater consistency is the relatively higher values of the *k_Fy_* vs. *F_xm_*, *F_zm_*, and *F_ym_* (Table 1). As for *k_Fx_*, also for *k_Fy_*, the greatest coherence occurred in terms of the *F_ym_* lateral force, but the correlation coefficient was negative, r = −0.397. The values of the *k_Fy_* directional coefficients were quite high and even when the tools were working in the soil without stones, they were in the range 1–5 (Figure 12a), which means that the angles of deviation for resultant force *F_m_* in relation to the *xz* vertical plane were in the range of 45–79°. The directional coefficients *k_Fy_* of the *F_m_* force for flexible and stiff tines working at a depth of 0.07 m in soil with stones vs. soils without stones increased by 119 and 35%, respectively, and showed a downward trend vs. working speed (Figure 12). These changes in the directional coefficients indicate the aforementioned asymmetry in the load of the duckfoot attached to these tines.

Interestingly, in this context, the direction of the resultant force *F_m_* in the vertical plane *xz* for the coulters working both on soil without stones and with stones did not change significantly also in terms of speed and working depth and amounted to 0.72–1.04 (Figure 12). The resultant force *F_m_* with in the *xz* vertical plane was inclined at angles of 36–46°.

**Figure 13:**
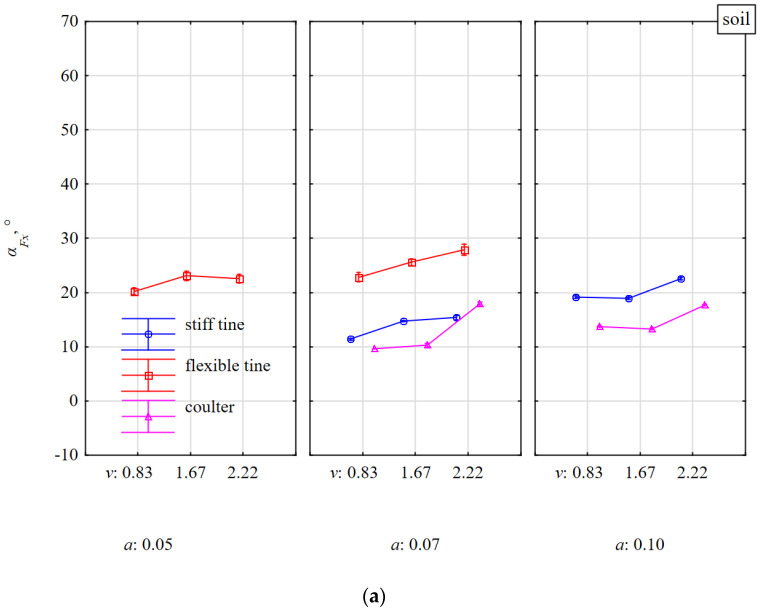

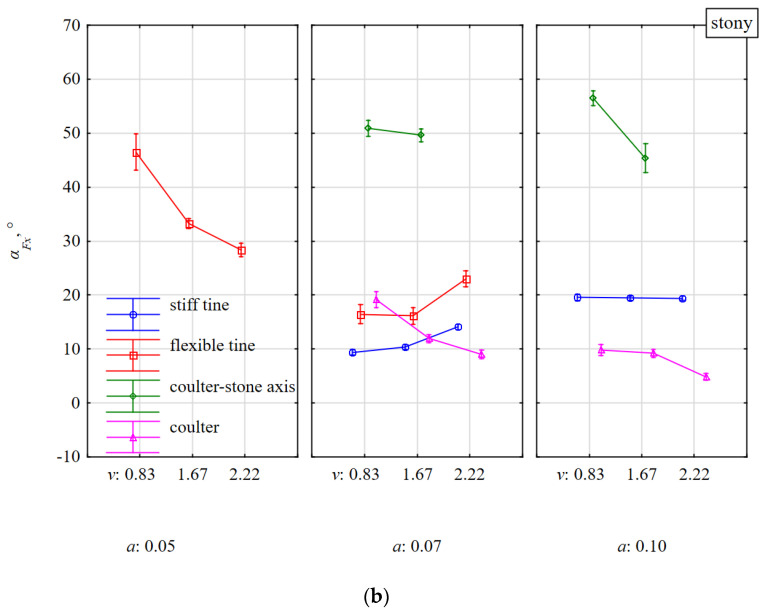
The tilt angle *α_Fx_* of resultant force *F_m_* in the 3D plane, in relations to the horizontal plane *xy*, affecting tools working at different depths *a* and working speeds *v* in the soil without stones bez (**a**) and with stones (**b**).

The modulus of the tilt angle *α_Fx_* of the resultant force *F_m_* in the 3D plane was to the greatest extent consistent with the vertical specific force *F_zm_*, as the correlation coefficient between these parameters was −0.613 (Table 1). Therefore, it was a negative high correlation. Contrary to the directional coefficient *k_Fx_* (Figure 10), the angle *α_Fx_* of the resultant force *F_m_* (Figure 13) because of the symmetry property of the modulus allowed to determine the absolute direction of the tool reaction. This remark applies mainly to the duckfoot attached to the flexible and stiff tines when the impact of the tool with the stone was not halfway along its length and the force *F_m_* was directed in different directions in the 3D plane (Figure 10, Figure 11 and Figure 12).

The greatest changes in the tilt angles *α_Fx_* of the resultant force *F_m_* occurred during the work of flexible tine in soil with stones vs. without stones for a working depth of 0.05 m and these angles decreased with the working speed (Figure 13). These changes in tilt angles indicate the aforementioned asymmetry of the tool’s impact with stones or of the attachment of the duckfoot to the tine.

The smallest tilt angles *α_Fx_* (4.8°–19.2°) of the resultant force *F_m_* were characterized by the work of the coulter covering the stone. The *α_Fx_* angles decreased with the speed and working depth (Figure 13). During the work of the coulter impacting its edge with the stone, the tilt angles *α_Fx_* of the resultant force *F_m_* were the highest (45.4°–56.5°) and decreased with the working speed, but the average values for the working depths of 0.07 and 0.10 m were the same (about 50°). The high value of these angles *α_Fx_* was determined by the specific vertical forces *F_zm_* (Figure 8b).

## 4. Conclusions

The kinetics and dynamics of stiff and flexible tines with a 135 mm wide duckfoot and a coulter with a diameter of 560 mm and an rake angle of 23° after an impact with silicon stones embedded in compacted soil (con index = 421 kPa) with sphericity 0.79–0.90 and mass of 2.53–4.72 kg were examined. The beak of the duckfoot was positioned in the axis of the row of five stones embedded in the soil at the depth of their thickness at a distance of 0.80 m. The coulter covered the stone or impact the edge of the stone halfway along its length. The tools worked at a speed of 0.83–2.22 m·s^−1^ and a working depth of 0.05–0.10 m. The results of specific parameters were compared to the response of the tools to loads in soil without stones. For both soil conditions, the kinetics of the flexible tine was the most reactive and the dynamic loads were lower than for the stiff tine. During the impact of the flexible tine with the stone, the specific accelerations in the direction of movement and vertical were 34.9 and 28.6 m·s^−^^2^·kg^−^^1^, respectively, and of a stiff tine (directed downwards) were −12.2 and −1.3 m·s^−^^2^·kg^−^^1^, respectively. Under these operating conditions, the specific forces (directed downwards) for the flexible tine were 229 and –124 N·kg^−^^1^, respectively, and for the stiff tine were 449 and 114 N·kg^−^^1^, respectively. Flexible tine impact with stone vs. soil without stones generated more than twice the kinetic reaction in the direction of movement and in the vertical direction. The stiff tine in the direction of movement achieved almost seven times greater accelerations than the forces and in the vertical direction these values were similar for both operating states of the tool. The reactions of both tines were suppressed with the working depth because of the more favorable impact place of the duckfoot beak with the stone. Along with the working speed, the kinetic and dynamic reactions of the flexible tine tended to increase. For the stiff tine, the specific accelerations decreased significantly with the working speed and the specific forces increased slightly.

Among the two systems of setting the coulter in relation to the stones, the impact of the cutting edge of the coulter with the stone in the middle of its length was more unfavorable than the work of the coulter covering the stone. During the impact, the edge of the coulter pressed the stone into the soil generating a significant increase in forces and specific accelerations vs. coulter work in soil without stones. The forces on the coulter pressing the stone into the soil were higher the greater the depth and working speed were because of the greater distance of the stone and the dynamics of the impact, respectively. For all tools, the directions of the lateral kinetic and dynamic responses were random, depending on the place of the impact of duckfoot beak or the cutting edge of the coulter with the stone.

The test results can be useful for improving the design of working tools by determining their geometry, taking into account modern materials and thermo-chemical treatments during their manufacture [22]. In the future, the determination of kinetic and dynamic responses of tools generated during an impact with a stone may be based on calculations using the finite element method.

## Figures and Tables

**Table 1:** Matrix of values of correlation coefficients for factors and parameters characterizing the kinetics and dynamics of movement of tools working in soil without stones and with stones at various speeds and working depths.

Parameter	*tool*	*i_s_*	*a*	*v*	*m_s_*	*row*	*a_zm_*	*a_ym_*	*F_xm_*	*F_zm_*	*F_ym_*	*k_Fxy_*	*k_Fx_*	*k_Fy_*	*α_Fx_*
*tool*	1.000														
*i_s_*	0.142 ^a^	1.000													
*a*	0.145 ^a^	0.080 ^a^	1.000												
*v*	−0.021	−0.008	−0.017	1.000											
*m_s_*	0.008	0.057 ^a^	0.005	−0.001	1.000										
*row*	0.204 ^a^	0.065 ^a^	0.248 ^a^	0.065 ^a^	0.004	1.000									
*a_zm_*	−0.107 ^a^	−0.131 ^a^	−0.573 ^a^	0.054 ^a^	−0.149 ^a^	−0.279 ^a^	1.000								
*a_ym_*	0.020	−0.049 ^a^	−0.477 ^a^	−0.003	−0.092 ^a^	−0.150 ^a^	0.759 ^a^	1.000							
*F_xm_*	−0.235 ^a^	−0.095 ^a^	0.209 ^a^	0.116 ^a^	−0.220 ^a^	0.217 ^a^	−0.370 ^a^	−0.290 ^a^	1.000						
*F_zm_*	−0.356 ^a^	−0.070 ^a^	0.078 ^a^	0.130 ^a^	−0.018	−0.310 ^a^	−0.147 ^a^	−0.202 ^a^	−0.218 ^a^	1.000					
*F_ym_*	0.772 ^a^	0.184 ^a^	0.237 ^a^	0.069 ^a^	−0.039 ^a^	0.228 ^a^	−0.249 ^a^	−0.093 ^a^	0.092 ^a^	−0.392 ^a^	1.000				
*k_Fxy_*	0.807 ^a^	0.184 ^a^	0.092 ^a^	−0.019	0.007	0.159 ^a^	−0.153 ^a^	−0.018	−0.284 ^a^	−0.177 ^a^	0.775 ^a^	1.000			
*k_Fx_*	−0.022	−0.025	0.177 ^a^	0.128 ^a^	−0.038 ^a^	−0.052 ^a^	−0.082 ^a^	−0.062 ^a^	0.184 ^a^	0.132 ^a^	0.044 ^a^	−0.190 ^a^	1.000		
*k_Fy_*	−0.446 ^a^	−0.084 ^a^	−0.110 ^a^	−0.128 ^a^	−0.163 ^a^	−0.149 ^a^	0.054 ^a^	−0.072 ^a^	0.155 ^a^	0.296 ^a^	−0.397 ^a^	−0.463 ^a^	0.118 ^a^	1.000	
*α_Fx_*	−0.095 ^a^	0.047 ^a^	−0.245 ^a^	−0.158 ^a^	0.148 ^a^	0.168 ^a^	0.219 ^a^	0.178 ^a^	−0.033 ^a^	−0.613 ^a^	−0.151 ^a^	−0.046 ^a^	−0.442 ^a^	−0.374 ^a^	1.000

*Tool*; statistic cods for tools: stiff tine—101, flexible tine—102, coulter-stone axis—103, coulter—104; *i_s_*—number of stones in the row; *a*—working depth; *v*—working speed; *m_s_*—mass of stone; *row*—stone row number; *a_ym_* and *a_zm_*—specific accelerations: lateral and vertical, respectively; *F_xm_*, *F_ym_* and *F_zm_*—specific forces: draught, lateral and vertical, respectively; *k_Fxy_*—the directional coefficient of the specific resultant force *F_m_* in the horizontal plane *xy*; *k_Fx_*, and *k_Fy_*—the directional coefficient of the specific resultant force *F_m_* in the 3D plane, relative to the resultant force on the horizontal and lateral planes; *α_Fx_* –modulus of the tilt angle of resultant force *F_m_* in the 3D plane, relative to the resultant force in the *xy* horizontal plane. ^a^—statistically significant correlation at *p* = 0.05.

**Table 2:** Mean values and standard deviations of parameters characterizing the kinetics and dynamics of movement of tools working in soil without stones and stony at various speeds and working depths.

Factor	*a_zm_*, m·s^−2^·kg^−1^	*a_ym_*, m·s^−2^·kg^−1^	*F_xm_*, N·kg^−1^	*F_zm_*, N·kg^−1^	*F_ym_*, N·kg^−1^	*k_Fxy_* −	*k_Fx_*, −	*k_Fy_*, −	*α_Fx_*, °
*p*-value									
*Tool*	<0.0001	<0.0001	<0.0001	<0.0001	<0.0001	<0.0001	<0.0001	<0.0001	<0.0001
*z_s_*	<0.0001	0.0332	<0.0001	<0.0001	0.9636	0.0120	<0.0001	0.0009	0.0461
*a*	<0.0001	<0.0001	<0.0001	<0.0001	<0.0001	<0.0001	<0.0001	<0.0001	<0.0001
*v*	<0.0001	<0.0001	<0.0001	<0.0001	<0.0001	<0.0001	<0.0001	<0.0001	<0.0001
Mean ± SD for type of tools									
Stiff tine	−1.13 ^c^ * ± 2.01	−1.54 ^c^ ± 1.96	443 ^c^ ± 175	111 ^d^ ± 42	18 ^a^ ± 38	0.04 ^a^ ± 0.07	0.28 ^c^ ± 0.12	4.23 ^c^ ± 2.4	15.2 ^b^ ± 6.1
Flexible tine	27.43 ^a^ ± 12.41	9.60 ^a^ ± 7.01	225 ^a^ ± 79	−111 ^b^ ± 98	43 ^b^ ± 48	0.24 ^b^ ± 0.32	−1.41 ^a^ ± 6.49	3.19 ^b^ ± 4.21	26.6 ^c^ ± 19.2
Coulter	1.16 ^b^ ± 0.33	0.93 ^b^ ± 0.75	301 ^b^ ± 73	−75 ^c^ ± 132	315 ^d^ ± 82	0.80 ^d^ ± 0.09	−0.16 ^b^ ± 0.24	0.91 ^a^ ± 0.14	10.9 ^a^ ± 9.3
Coulter-stone axis	1.77 ^b^ ± 1.18	1.46 ^b^ ± 1.59	580 ^d^ ± 191	−836 ^a^ ± 454	236 ^c^ ± 304	0.29 ^c^ ± 0.29	−1.3 ^a^ ± 0.41	0.71 ^a^ ± 0.18	50.6 ^d^ ± 9.7
Mean ± SD for soil condition—*z_s_*									
Soil	4.37 ^b^ ± 4.36	2.26 ^b^ ± 2.35	257 ^a^ ± 101	93 ^b^ ± 45	129 ^a^ ± 179	0.30 ^a^ ± 0.36	0.33 ^b^ ± 0.10	2.16 ^a^ ± 1.13	18.2 ^a^ ± 5.3
Stony	9.11 ^a^ ± 14.95	2.98 ^a^ ± 6.41	344 ^b^ ± 167	−85 ^a^ ± 268	128 ^a^ ± 161	0.35 ^b^ ± 0.37	−0.54 ^a^ ± 3.84	2.71 ^b^ ± 3.16	19.8 ^b^ ± 16.7
Mean ± SD for depth *a* (m)									
0.05	28.45 ^a^ ± 12.11	9.44 ^a^ ± 6.86	205 ^a^ ± 89	−138 ^a^ ± 86	39 ^a^ ± 34	0.30 ^a^ ± 0.42	−2.65 ^a^ ± 9.30	1.83 ^a^ ± 1.75	35.1 ^b^ ± 19.9
0.07	8.70 ^b^ ± 14.41	3.15 ^b^ ± 6.39	362 ^b^ ± 176	−69 ^b^ ± 209	128 ^b^ ± 153	0.33 ^b^ ± 0.36	−0.17 ^b^ ± 0.45	3.47 ^c^ ± 3.93	16.7 ^a^ ± 14.0
0.10	0.19 ^c^ ± 1.71	0.33 ^c^ ± 1.71	364 ^b^ ± 143	−56 ^c^ ± 368	170 ^c^ ± 192	0.40 ^c^ ± 0.37	−0.01 ^b^ ± 0.52	1.83 ^a^ ± 1.07	17.4 ^a^ ± 13.9
Mean ± SD for speed *v* (m·s^−1^)									
0.83	7.61 ^a^ ± 12.01	2.63 ^b^ ± 5.79	303 ^a^ ± 128	−102 ^a^ ± 247	108 ^a^ ± 137	0.35 ^b^ ± 0.41	−1.17 ^a^ ± 6.30	3.27 ^b^ ± 4.35	22.7 ^c^ ± 20.9
1.67	9.57 ^b^ ± 15.61	3.62 ^a^ ± 7.04	371 ^c^ ± 204	−110 ^a^ ± 345	146 ^c^ ± 183	0.36 ^c^ ± 0.34	−0.20 ^b^ ± 0.49	2.37 ^a^ ± 2.25	19.4 ^b^ ± 14.2
2.22	9.38 ^b^ ± 15.9	2.54 ^b^ ± 5.75	344 ^b^ ± 146	−7 ^b^ ± 132	131 ^b^ ± 162	0.33 ^a^ ± 0.36	−0.08 ^b^ ± 0.39	2.37 ^a^ ± 1.88	16.8 ^a^ ± 11.3

*Tool* (stiff tine, flexible tine, coulter, coulter-stone axis); *z_s_* –soil condition; *a*—working depth; *v*—working speed; *a_ym_* and *a_zm_*—specific accelerations: lateral and vertical, respectively; *F_xm_*, *F_ym_* and *F_zm_*—specific forces: draught, lateral and vertical, respectively,; *k_Fxy_*—the directional coefficient of the specific resultant force *F_m_* in the horizontal plane *xy*; *k_Fx_*, and *k_Fy_*—the directional coefficient of the specific resultant force *F_m_* in the 3D plane, relative to the resultant force on the horizontal and lateral planes; *α_Fx_* –modulus of the tilt angle of resultant force *F_m_* in the 3D plane, relative to the resultant force in the *xy* horizontal plane. * Different letters in the columns (a, b, c, d) for the same factor indicate a statistically significant difference between the mean values.

## Data Availability

Not applicable.

## Nomenclature

*a*	working depth of tool (m),
*a_xm_*, *a_ym_*, *a_zm_*	specific acceleration: draught, lateral and vertical, respectively (m·s^−^^2^·kg^−1^),
*F_m_*	specific resultant force in the 3D plane (N·kg^−1^),
*F_x_*, *F_y_*, *F_z_*	component forces: draught, lateral and vertical, respectively (N),
*F_xm_*, *F_ym_*, *F_zm_*	specific forces: draught, lateral and vertical, respectively (N·kg^−1^),
*F_xym_*	resultant specific force in the horizontal plane *xy* (N·kg^−1^),
*i_s_*	number of stones in the row (−),
*k_Fx_*, *k_Fz_*, *k_Fy_*	the directional coefficient of the specific resultant force *F_m_* in the 3D plane, relative to the resultant force on the *xy* horizontal plane, *xz* vertical plane (face), *yz* vertical frontal plane (−),
*k_Fxy_*	the directional coefficient of the specific force *F_xym_* in the horizontal plane *xy* (−),
*MC*	moisture content of the soil relative to the wet substance (%),
*m_s_*, *m_e_*	*mass of stone and mass of soil with a volume of stone, respectively (kg)*,
*row*	stone row number (−),
*v*	working speed of the tool (m·s^−1^),
*z_s_*	soil condition (−),
*α_Fx_*, *α_Fz_*, *α_Fy_*	modulus of the tilt angle of resultant force *F_m_* in the 3D plane, relative to the resultant force in the *xy* horizontal plane, *xz* vertical plane (face) and *yz* vertical frontal plane, respectively (°),
*α_Fxy_*	modulus of the tilt angle of force in the *xy* horizontal plane (°),
*ρ_s_*, *ρ_e_*	densities of stone and DM of soil, respectively, (kg·m^−3^).
